# Outlines of Human Physiology

**Published:** 1839-07

**Authors:** 


Art. IV.—*Outlines of Human Physiology.* By William Pulteney Alison, m.d. f.r.s.e., Professor of the Institutes of Medicine in the University of Edinburgh, &c. *Third Edition.—Edinburgh*, 1839. 8vo, pp. 457.

We welcome the appearance of a new edition of this valuable work, for two reasons: first, because we think it a pity that a treatise of such acknowledged excellence should be allowed to remain in the rear of the rapidly-advancing march of physiological science; and, second, because it speaks well for the improving taste of the students of this country that a steady demand should exist for a work of so abstract yet at the same time philosophical a character. Dr. Alison's treatise is not one which will assist those who wish to gain a mere smattering of physiology. It embodies all the generalizations which he regards as well established; together with such hypotheses as may assist, if properly employed, in the attainment of truth; but for a mass of individual facts the student must look elsewhere. This plan has its advantages, especially in a work intended principally as a text-book for lectures; and we cannot imagine one better calculated to excite enquiry in the minds of an industrious student, and to induce him to seek for information from other and more scattered sources, whilst, at the same time, it exercises his discrimination in applying the laws here laid down to the facts which he meets with in his researches. But we are afraid that the book will never find favour with those who need to be *attracted* to the study of a science which appears to us to be unsurpassed in interest. It contains no showy novelties, nor brilliant speculations. It affords, however, a most solid foundation for advantageous study; not only in the scientific value of its contents, but in the philosophical spirit which pervades it, and which impresses itself upon its readers.

In the present edition the Supplement, which was published by Dr. A. about three years since, is incorporated with the text. A most valuable addition which is thus made, is the chapter on the general laws and conditions of vital action, with which the treatise now commences. In this, the author states himself to have endeavoured "to define, with as much precision as the present state of our knowledge permits, the nature of those vital properties or powers to which we must refer as the agents immediately concerned in producing the phenomena of life...... Using these terms only as general expressions for the unknown causes of phenomena, which have been studied, classified, and generalized, and which are peculiar to the living state, I confidently maintain that the reference to such ultimate facts or general principles in physiology is perfectly in accordance with the rules of true philosophy." The only danger in such a course seems to be that we may be too easily satisfied with referring to such "ultimate facts" for an easy explanation of phenomena, which the extending grasp of the physical sciences may be hereafter found to include. There is a large class of phenomena to which the term *vital* can only be provisionally applied; whilst there are unquestionably others completely beyond the pale of dynamics and chemistry, on which the science of vitality, expressing its peculiar powers and actions, must be based. We are not yet in a state to separate these with certainty, and a premature attempt will be more injurious than beneficial.

The results of many recent physiological enquiries will be found embodied in this volume, which is, in almost every respect, *au courant* with the present state of the science. To the student we would particularly mention the subjects of muscular contraction, and of nutrition and secretion, considered in reference to nervous influence; and also that of the capillary circulation, as deserving his careful attention in reading this volume. Dr. Alison's views on these points, though opposed to those of most other physiologists, appear to us equally profound and well supported; and we hope to see them gain ground amongst the rising generation, though opposed to some antiquated prejudices. We cannot, then, do more than strongly recommend his work to all industrious and enquiring students, as well as to those who are desirous of adapting their knowledge of *principles* to the advanced state of the science; the only mode of keeping pace with it open to those who are actively engaged in the practical duties of their profession.

